# SIRT1/PARP1 crosstalk: connecting DNA damage and metabolism

**DOI:** 10.1186/2041-9414-4-6

**Published:** 2013-12-20

**Authors:** Augustin Luna, Mirit I Aladjem, Kurt W Kohn

**Affiliations:** 1 Laboratory of Molecular Pharmacology, Center for Cancer Research, National Cancer Institute, NIH, Bethesda, MD 20892, USA; 2 Bioinformatics Program, Boston University, Boston, MA 02215, USA

**Keywords:** SIRT1, Sirtuins, PARP1, Poly-(ADP) polymerases, Metabolism, Nicotinamide Adenine Dinucleotide (NAD+) competition, Post-translational modifications, Transcriptional regulation, DNA damage repair, Circadian rhythms

## Abstract

An intricate network regulates the activities of SIRT1 and PARP1 proteins and continues to be uncovered. Both SIRT1 and PARP1 share a common co-factor nicotinamide adenine dinucleotide (NAD+) and several common substrates, including regulators of DNA damage response and circadian rhythms. We review this complex network using an interactive Molecular Interaction Map (MIM) to explore the interplay between these two proteins. Here we discuss how NAD + competition and post-transcriptional/translational feedback mechanisms create a regulatory network sensitive to environmental cues, such as genotoxic stress and metabolic states, and examine the role of those interactions in DNA repair and ultimately, cell fate decisions.

## Introduction

SIRT1 and PARP1 are enzymes that affect two key post-translational modifications: acetylation and ADP-ribosylation, respectively, for a diverse group of proteins. These enzymes are functionally connected due to their use of a common substrate, nicotinamide adenine dinucleotide (NAD+) [1,2]. Recent studies suggest that these proteins participate in common pathways providing cells with a mechanism for balancing cell survival and death. A well-developed understanding of activity overlap of these proteins may provide insights into the biology of these two proteins as they are actively being pursued as therapeutic targets in a range of conditions, including cancer and metabolic disorders [3-5].

**Figure 1:**
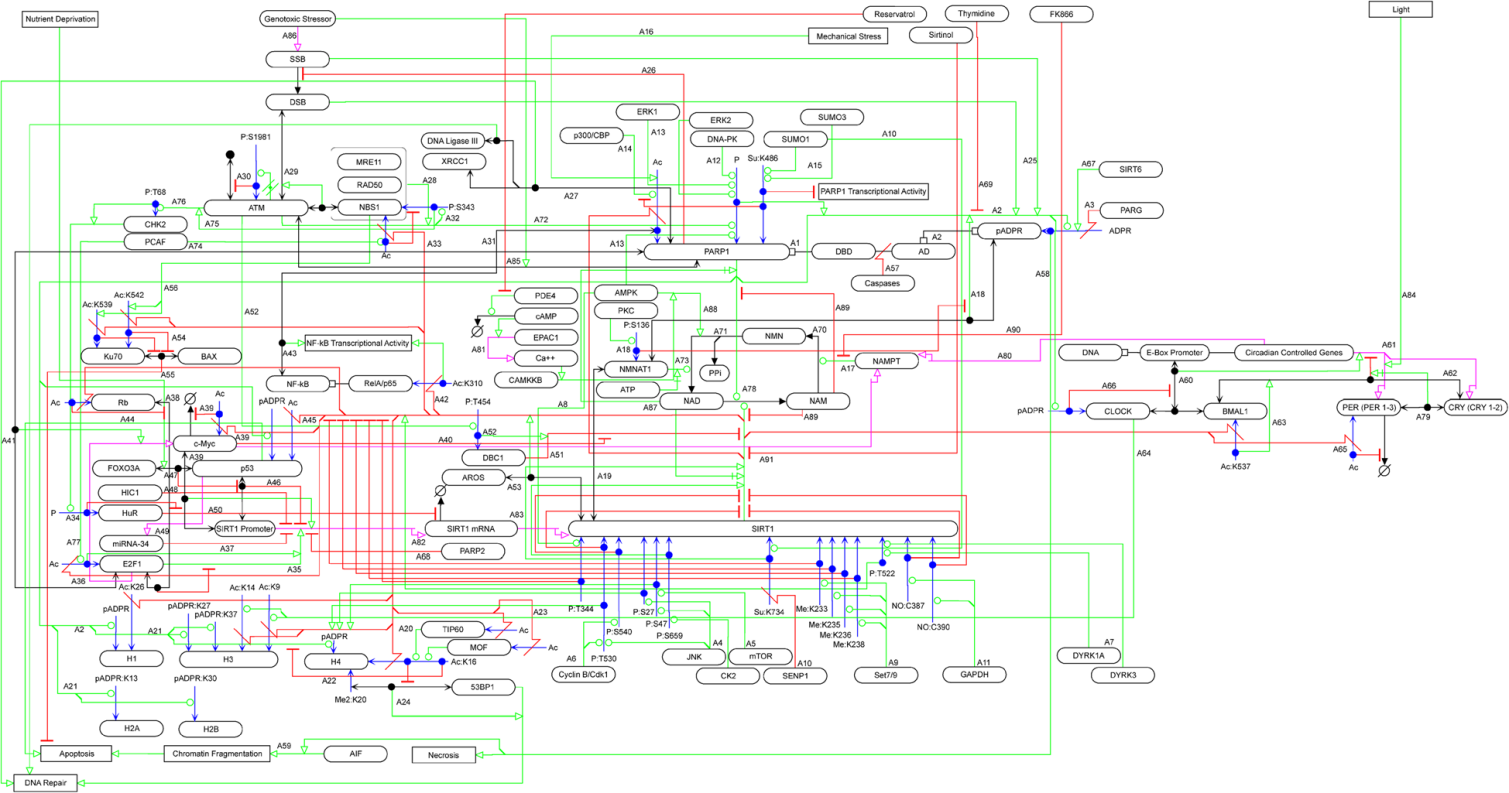
**SIRT1 and PARP1 Molecular Interaction Map.** Blue: Post-translational modifications, Red: Inhibition and cleavage, Green: Stimulation and catalysis.

In this review, we look at the role of each of these two proteins using a Molecular Interaction Map (MIM) that visually integrates the experimental findings of the regulatory pathways that surround these proteins, shown in Figure 1. The MIM helps free readers from a linear view of events and gain a better understanding of control loops involved in these pathways [6]. A machine-readable version of the MIM is provided as Additional file 1 viewable using PathVisio-MIM ( http://discover.nci.nih.gov/mim/mim_pathvisio.html) [7]. Additionally, the MIM covers in greater detail the interactions surrounding SIRT1 and PARP1; a complete list of annotations is also provided as Additional file 2.

**Figure 2:**
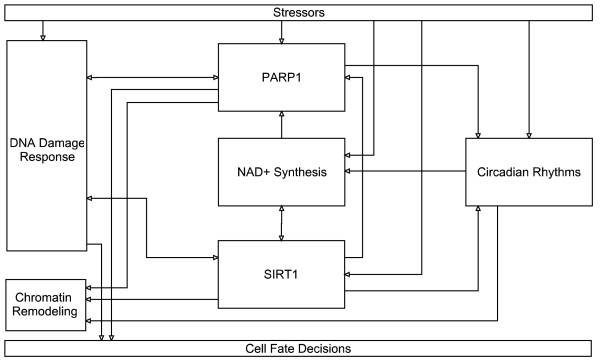
**Modular layout diagram of SIRT1 and PARP1 MIM.** Arrows connecting modules indicate that a node starting in a module connects to a node in the target module.

**Figure 3:**
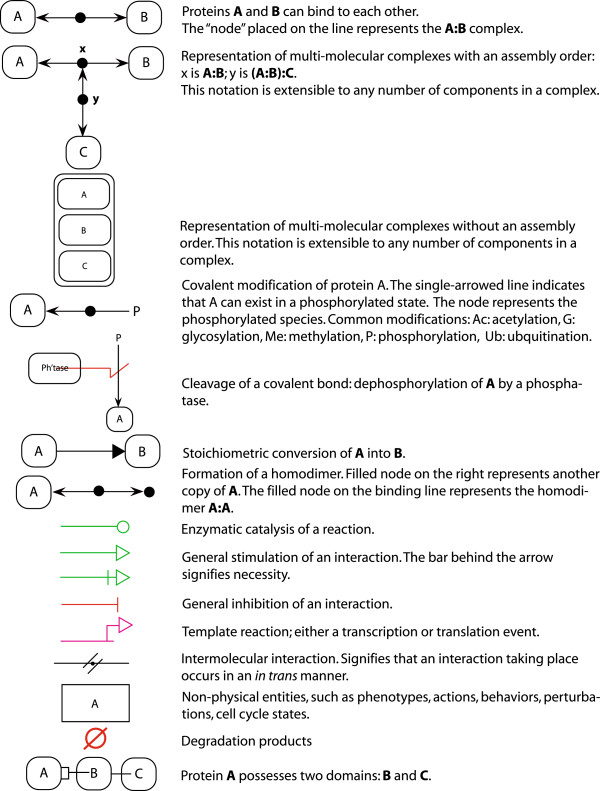
Reference guide for the MIM notation.

Figure 2 shows a modular overview of how the SIRT1 and PARP1 interactions are laid out (an arrow indicates that a molecule or process from the source module has an interaction with a molecule or process in the target module) and Figure 3 provides a legend for reading the MIM notation. Throughout this review readers will see annotation labels in double square brackets and prefixed with a letter that refer to specific interactions in the MIM shown in Figure 1 and Additional file 2. We focus on aspects that alter the activity of these proteins, including: post-translational modifications, co-regulation, NAD + competition and co-regulated targets. Additionally, we discuss outstanding questions that would more accurately describe the relationship between these proteins.

## Review

### SIRT1

Sirtuins were originally discovered in yeast where the SIR (Silent Information Regulator) genes are necessary for the repression of silent mating-type loci [1]. The mammalian family of sirtuins consists of 7 proteins, SIRT1-7, which are ubiquitously expressed. Three of the sirtuins, SIRT1, SIRT6, and SIRT7 localize to the nucleus, SIRT2 is mainly localized in the cytoplasm, while the remaining three sirtuins: SIRT3, SIRT4, and SIRT5 are found in the mitochondria [8]. The members of the sirtuin family not only differ in cellular locations, but also in enzymatic function. SIRT1 and SIRT5 are primarily protein deacetylases, SIRT4 and SIRT6 are mono(ADP)-ribosyltransferases, and SIRT2 and SIRT3 exhibit both enzymatic activities; no clear functionality has been attributed to SIRT 7 [9-14]. The conserved catalytic domain of sirtuins is capable of carrying out both deacetylation and ADP-ribosylation activities using NAD+, and it has been suggested that sirtuins may have the potential to carry out either enzymatic activity under the right conditions [15,16]. This review will focus on SIRT1 (EC 3.5.1.-) a member of the sirtuin family that is expressed in many tissues and acts as a NAD + -dependent protein deacetylase [1]. SIRT1 has been implicated in signaling pathways underlying various diseases, including: diabetes, cardiovascular disease, neurodegeneration, cancer, aging, and obesity [17].

### PARP1

PARP1 (EC 2.4.2.30) is an NAD + -dependent nuclear ADP-ribosyltransferase with three domains: a DNA binding domain (DBD), an auto-modification domain (AD), and a catalytic domain [[A1]] [18]. The PARP family of proteins are involved in many processes, including: DNA damage response, cell death, cell cycle regulation, and telomere regulation [19]. The main function of PARP1 is the formation of poly(ADP-ribose) (PAR) chains on itself and other proteins [[A2]] [2,20,21]. PARP1 is a transcriptional co-activator where PAR acts as a signal helping to regulate transcription [22]. PAR is quickly cleaved by poly (ADP-ribose) glycohydrolase (PARG) [[A3]] [20]. PARP1 becomes highly activated by DNA strand breaks; electrostatic repulsion between the poly (ADP)-ribose (PAR) chains and DNA eventually leads to its catalytic inactivation [18].

### Post-transcriptional regulation of SIRT1 and PARP1

Here we review post-translational modifications that affect the activities of SIRT1 and PARP1.

### SIRT1

For a comprehensive review of sirtuin modifications, see Flick and Luscher [23]. Below we describe several of these modifications for SIRT1 and augment this list with additional modifications.

SIRT1 phosphorylation results in both stimulatory and inhibitory effects. Phosphorylation of SIRT1 by JNK occurs at three sites: S27, S47, and T530 in response to oxidative stress that stimulates its deacetylation activity [[A4]] [24]. In contrast, mTOR also phosphorylates SIRT1 in response to oxidative stress, but only at a single site, S47, resulting in the inhibition of SIRT1 [[A5]] suggesting a multi-site phosphorylation regulatory mechanism is in place; such a mechanism may be involved in the regulation of the timing of SIRT1 activity [25,26]. CK2 phosphorylates human SIRT1 at S659 and S661, both *in vivo* and *in vitro*, stimulating its deacetylation activity [[A4]] [27,28]. These two phosphorylation sites exist in a region of SIRT1 that are essential for SIRT1 activity both for its catalytic activity and ability to bind to substrates [29]. Cyclin B/Cdk1, a cell cycle-dependent kinase, can phosphorylate SIRT1 at T530 and S540 [[A6]]. Phosphorylation at these two sites decreases the activity of SIRT1 and disrupts progress of the cell cycle [30]. Similar to the case with mTOR and S47, T530 is a site phosphorylated by JNK and may also function as a part of a combinatorial modification program.

Kinases DYRK1A and DYRK3 have been shown to phosphorylate human SIRT1 at T522, stimulating the deacetylation of p53 by SIRT1[[A7]]; phosphorylation at this site increases the rate of product release by SIRT1 [31]. AMPK phosphorylates human SIRT1 at T344 inhibiting its ability to decacetylate p53, a known target of SIRT1 [[A8]] [32]. In addition to phosphorylation, methylation of SIRT1 by Set7/9 at K233, K235, K236, and K238 inhibits the SIRT1-mediated deacetylation of p53 in response to DNA damage [[A9]] [33]. Sumoylation at K734 by SUMO1 increases, whereas desumoylation by SENP1 decreases, the activity of SIRT1 in response to genotoxic stress [[A10]] [34]. In this study, genotoxic stress promoted the association of SIRT1 with SENP1, which may help to inhibit the ability of SIRT1 to promote survival. Additionally, transnitrosylation of SIRT1 by GAPDH at C387 and C390 has been found to inhibit the activity of SIRT1 leading to decreased PGC1α transcriptional activity; PGC1α is an important regulator of metabolism and mitochondrial function [[A11]] [35].

### PARP1

The activity of PARP1 can be modulated via post-translational modifications, including phosphorylation, sumoylation, and acetylation. DNA-PK phosphorylates PARP1 though its effect is unknown [[A12]] [36]. Phosphorylation of PARP1 by AMPK has been shown to enhance its activity [[A13]] [37]. This stimulation of PARP1 by AMPK contrasts with the AMPK-mediated inhibition of SIRT1 and suggests one mechanism by which AMPK, a metabolic sensor able to regulate ATP-consuming pathways, may be capable of controlling cell survival given the roles of PARP1 and SIRT1 in response to DNA damage. ERK1/2 has also been shown to phosphorylate PARP1 in neuronal cells and to stimulate the activity of PARP1 in response to DNA damage; inhibition of ERK1/2 results in the inhibition of PARP1-mediated cell death [38]. PARP1 is acetylated by p300/CBP; this acetylation is involved in the activation of NF-κB by PARP1 [[A14]] [39]. PARP1 is sumoylated by SUMO1 and SUMO3 at K486 of PARP1's auto-modification domain. This modification inhibits the ability of p300 to acetylate PARP1 and inhibits the expression of genes that are transcriptionally targeted by PARP1 [[A15]] [40].

### Co-regulation of SIRT1 and PARP1

#### Cross-modification and transcriptional co-regulation

SIRT1 and PARP1 are transcriptionally and functionally interconnected [41-43]. In SIRT1-deficient mouse cardiomyocytes, Rajamohan et al. in 2009 found increased levels of PARP1 acetylation in response to mechanical stress, suggesting that SIRT1 can deacetylate PARP1 [[A16]] [44]. Whether this interaction occurs during genotoxic stress or other types of stresses remains an open question. No similar modification reaction has been seen on SIRT1 by PARP1 in response to DNA damage. However, SIRT1 is able to negatively regulate the PARP1 promoter, and the SIRT1 promoter has been shown to be under the influence of PARP2 [45,46].

### NAD + competition

Another key co-regulatory mechanism between these two proteins is the utilization of nicotinamide adenine dinucleotide (NAD+). It has been suggested by several studies that activation of PARP1 causes a depletion in NAD + levels, which inhibits SIRT1 activity [42-45]. In mammals, NAD + is mainly generated through the salvage pathway; this pathway involves nicotinamide (NAM) as the major precursor in this multi-step process that involves the conversion of NAM into nicotinamide mononucleotide (NMN) and then NMN into NAD+. The rate-limiting protein in the NAM-NMN-NAD + conversion is nicotinamide phosphoribosyltransferase (NAMPT) [[A17]]. PARP1 was shown to have a greater effect on NAD + depletion than SIRT1 in response to the NAMPT inhibitor, FK866 [47]. In a related study, the inhibition of NAMPT by FK866 was shown to produce an effect similar to SIRT1 depletion [48].

**Figure 4:**
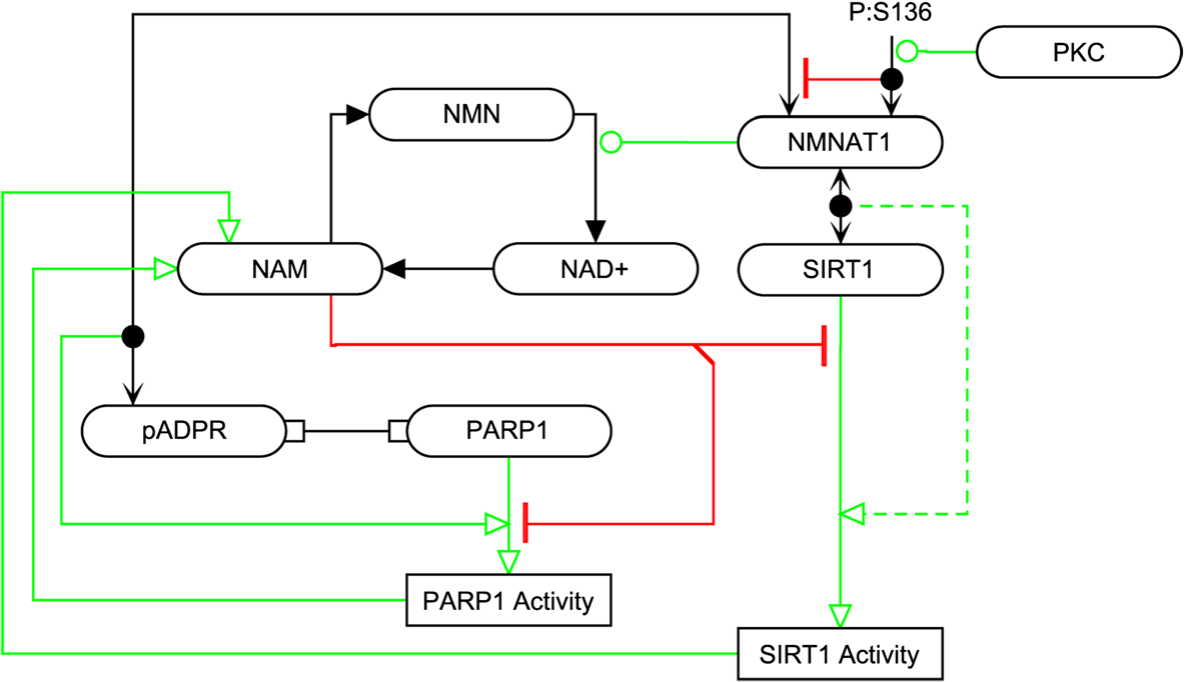
**The effect of NMNAT1 binding on SIRT1 and PARP1 activity.** Zhang et al. proposed that NMNAT1 may stimulate SIRT1 activity; indicated by the dashed line.

Recent evidence suggests that local supplies of NAD + may be important for the enzymatic activities of these two proteins, shown in Figure 4[49-51]. Nicotinamide mononucleotide adenylyltransferase 1 (NMNAT1), which catalyzes the conversion of NMN into NAD + in the synthesis of NAD + via the salvage pathway, can bind to (ADP-ribose) polymers in vitro leading to the stimulation of PARP1 activity; this effect is diminished when NMNAT1 is phosphorylated at S136 by protein kinase C (PKC) [[A18]] [52]. A similar interaction has been shown to occur with SIRT1, whereby SIRT1 binds to NMNAT1 helping to recruit NMNAT1 to specific promoters, which may help stimulate SIRT1 activity [[A19]] [53]. PARP1 activity leads to increased NAM concentrations at DNA damage sites, potentially leading to local inhibition of SIRT1 histone deacetylase activity [54]. Further work is needed to understand the function of these two proteins around chromatin sites occupied beforehand by one of them.

### Regulation of common SIRT1 and PARP1 targets

#### SIRT1/PARP1 response to DNA damage

DNA damage response to both endogenous and exogenous sources is an intricate process that is not fully understood due to the complexity of the potential lesions, the number of proteins involved in both surveillance and repair, the interconnected regulation of proteins involved in the detection and repair of damage, stoppage of the cell cycle, and the potential induction of cell death; reviewed by [55]. SIRT1 and PARP1 play several roles throughout the response to DNA damage from the initial response to final cell fate decisions.

#### Histones

Both SIRT1 and PARP1 are known to modify histones; deacetylation of histones triggers chromatin compaction and the inhibition of transcription, whereas poly (ADP-ribose) polymers help to relax chromatin. SIRT1 is capable of deacetylating several histone amino acid residues, including H1K26, H3K9, H3K14, and H4K16 [[A20]] [56,57], while PARP1 can modify histones H1, H2AK13, H2BK30, H3K27, H3K37, and H4K16 to possibly regulate transcription [[A21]] [21,58]. It has been suggested that ADP-ribosylation of histone H1 promotes transcription by inhibiting the ability of histone H1 to bind to DNA [59]. Additionally, a competitive interaction has been shown between acetylation and PAR where acetylation of H4K16 inhibits the ADP-ribosylation of histone H4 [[A22]] [21]. Here a potential contradiction in the role of SIRT1 in condensing chromatin arises whereby SIRT1 deacetylation activity could potentially help drive the PARP1 ADP-ribosylation activity on H4K16. Currently, it is known that SIRT1 plays a role in DNA damage repair via histone deacetylation through the deacetylation of the two histone acetyltransferases, TIP60 and MOF, which are able to acetylate histone H4 [[A23]]. Deacetylation of these two proteins promotes their ubiquitin-dependent degradation affecting DNA double-strand break (DSB) repair either through the repression of repair or affecting the choice of repair mechanism (i.e. homologous recombination or non-homologous end joining (NHEJ)) [60-63]. TIP60-dependent acetylation of H4K16 inhibits the binding of 53BP1 to H4K20me2, which promotes non-homologous end joining [[A24]] [62,64]. Further studies are needed to understand how DNA damage might affect modifications on the various histones though it is known that genotoxic stress causes a random redistribution of SIRT1 across the genome with a correlated increase in levels of H1K26 acetylation [65]. However, while PARP1 does localize to DNA strand breaks, it is also not known whether there is any further global redistribution PARP1 or a relationship to the redistribution of SIRT1.

#### DNA damage signaling pathway

Both SIRT1 and PARP1 are DNA damage responders and the absence of either of these proteins may lead to DNA damage sensitization [66,67]. PARP1 begins to localize to DNA breaks rapidly and becomes activated by binding to DNA breaks. The ADP-ribosylation activity of PARP1 increases 10–500 fold as a result of binding to DNA breaks [[A25]] [2]. Once activated, PARP1 may help repair single strand DNA breaks, preventing their conversion to double-stranded breaks [[A26]] [68]. In addition, PARP1 is involved in DNA repair through its associations with base excision repair (BER) enzymes such as polymerase β, XRCC1 and DNA ligase III by helping these proteins localize to sites of DNA damage [[A27]] [69].

Two early responders of DNA damage linked to SIRT1 and PARP1 regulation are ATM (ataxia telangiectasia, mutated) and CHK2 (checkpoint kinase 2) [70]. The activation of ATM by DNA breaks requires the activation of the MRE11-RAD50-NBS1 (MRN) complex [[A28-A30]]. It has been shown that PARP1 binds to ATM, an interaction that is stimulated by DNA damage, and that the automodification of PARP1 leads to ATM activation [[A31]] [67].

An extended feedback loop has been proposed by Gorospe and de Cabo involving SIRT1 and several key DNA damage repair proteins [70]. In this loop, NBS1 is phosphorylated by ATM in response to genotoxic stress at S343 for the activation of NBS1; to be phosphorylated, it is necessary for NBS1 to be in a hypoacetylated state, which SIRT1 helps to maintain by deacetylating NBS1 [[A32-A33]] [71,72]. CHK2 is activated when T68 is phosphorylated by ATM. CHK2 can then phosphorylate HuR at several sites causing it to dissociate from SIRT1 mRNA, and thereby reduce the half-life of the SIRT1 mRNA [[A34]] [73]. It has been suggested that in repairable DNA damage situations SIRT1 levels are elevated leading to a survival response, but during lethal DNA damage SIRT1 levels can be attenuated by CHK2 through the phosphorylation of HuR that can ultimately result in cell death [70].

**Figure 5:**
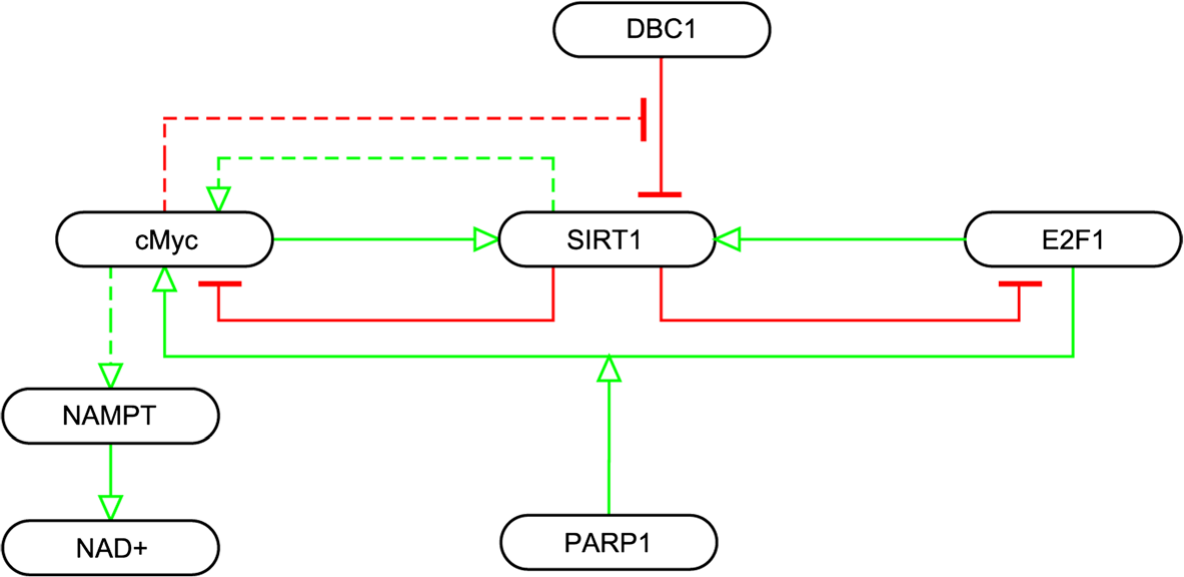
**Interactions between SIRT1 and transcription factors c-MYC and E2F1.** Solid lines between SIRT1 and c-MYC indicated are interactions from Yuan, Minter-Dykhouse et al. 2009 forming a negative feedback loop, while dashed lines are findings related to SIRT1 and c-MYC from Menssen, Hydbring et al. 2012.

SIRT1 is also regulated by c-MYC and E2F1, two proteins involved in cell proliferation, differentiation and apoptosis, through negative feedback loops shown in Figure 5. E2F1, a transcription factor, induces the transcription of SIRT1 [[A35]]. Conversely, E2F1 has been suggested to be a target for SIRT1 deacetylation, which inhibits E2F1 activity [[A36-A37]] [74]. Additionally, the transcriptional activity of E2F1 is inhibited by Retinoblastoma (Rb), which is another substrate of SIRT1 deacetylation; acetylation of Rb has been shown to regulate the binding of Rb to E2F1 [[A38]] [75,76]. Two studies have examined the interactions between SIRT1 and c-MYC producing contradictory results. In one publication, c-MYC over-expression leads to an increase in SIRT1 expression and then deacetylation of c-MYC by SIRT1 leads to the destabilization of c-MYC [[A39]] [77]. In the second publication, neither the induction of SIRT1 expression nor the destabilization of c-MYC was seen following c-MYC activation. Instead, a stabilizing effect on c-MYC due to deacetylation by SIRT1 was found. Also in the second study, Menssen et al. found that c-MYC can induce the transcription of NAMPT and help sequester DBC1, an inhibitor of SIRT1 [[A40]] [78]. Another line of evidence suggesting that SIRT1 may affect NAMPT through a second mechanism involving the circadian clock will be discussed later. There is evidence that PARP1 binds to E2F1 stimulating E2F1-dependent transcription of c-MYC [[A41]] [79]. This presents the possibility that both SIRT1 and PARP1 may be capable of influencing the regulation of NAMPT to affect NAD + levels through c-MYC.

Another co-regulated protein is NF-κB, a regulator of cellular response, including inflammation, to stress. In the case of NF-κB, the effects of SIRT1 and PARP1 are opposing. SIRT1 can deacetylate the RelA/p65 subunit of NF-κB at K310 to inhibit NF-κB transactivation activity [[A42]] [80]. PARP1 is an activator of NF-κB through its direct binding to NF-κB; acetylation of PARP1 by p300/CBP is required for the binding of PARP1 to NF-κB [[A43]] [39].

Given the importance of p53 to apoptotic response, a number of studies have focused on the regulation of p53 by SIRT1. p53 acts as a transcription factor that induces apoptosis and is inhibited by SIRT1 deacetylation [[A44]] [81,82]. SIRT1 has the capability of deacetylating p53 at several sites in mouse embryonic fibroblasts (MEFs) and SIRT1-deficient cells possess hyperacetylated p53; the precise role of p53 acetylation is unclear [[A45]] [83-85]. Several proteins help to modify the interactions of SIRT1 with p53, including p53 (itself), DBC1, AROS, and HIC1, suggesting that it is a cellular imperative to control the inhibition of p53 by SIRT1 under certain conditions. p53 can repress SIRT1 expression during nutrient abundance via p53-binding sites on the SIRT1 promoter. This effect is countered by the transcription factor FOXO3A, which interacts with p53 in an inhibitory fashion during nutrient deprivation [[A46-A47]] [86,87]. Hypermethylated in cancer-1 (HIC1) is a transcriptional repressor of the SIRT1 promoter [[A48]] that helps prevent age-dependent cancers in mice. If HIC1 is inhibited, SIRT1 expression increases, allowing for more efficient inactivation of p53; p53 over-expression leads to the transactivation of HIC1, thus creating a negative feedback loop [88].

Micro-RNAs have also been shown to downregulate SIRT1-dependent deacetylation of p53. p53 can stimulate the expression of miRNA-34 [[A49]], which subsequently drives down the expression of SIRT1 lowering SIRT1 availability to inhibit p53. Over 15 micro-RNAs affect the expression of SIRT1 either directly or by decreasing the expression of HuR, which stabilizes SIRT1 mRNA [[A50]] [89,90].

Given the well-studied nature of p53 as a SIRT1 substrate, p53 has been used to characterize SIRT1 inhibitors and activators. In humans, deleted in breast cancer 1 (DBC1) acts as an inhibitor of SIRT1 (an inhibitory effect increased by the phosphorylation of DBC1) and whose effect has been shown to lead to p53 hypoacetylation [[A51-A52]] [29,91-93]. Active Regulator of SIRT1 (AROS) has been shown to bind SIRT1 and help enhance the deacetylation of p53 by SIRT1 [[A53]] [94]. Further studies are needed to understand if the effects on p53 acetylation states are specific to the activities of DBC1 and AROS on SIRT1 or if other substrates of these two proteins are involved.

Much less is known about the interaction between PARP1 and p53. PARP1 helps p53 accumulate in the nucleus by (ADP-ribosyl)ating p53, which prevents p53 nuclear export [95], and there is evidence to suggest that SIRT1 deacetylation activity is capable of blocking p53 nuclear translocation [96].

#### Cell death

In addition to the role that SIRT1 plays in the inhibition of p53, SIRT1 is capable of deacetylating Ku70 at K539 and K542; the acetylation of Ku70 leads to the dissociation of the Ku70 and Bax helping to trigger apoptosis; Bax is a pro-apoptotic factor that is sequestered by Ku70 [[A54-A55]] [97]. NBS1, which we have discussed here beforehand as being activated via deacetylation by SIRT1, has also been shown to help control the interaction between Ku70 and Bax by stimulating the acetylation of Ku70 [[A56]] [98]. The exact conditions leading to a differential role of SIRT1 on the Ku70 and Bax complex remains to be uncovered.

PARP1 plays a role in cell death pathways (apoptosis or necrosis) in the course of responding to DNA damage. ATP is required for optimal caspase activation, and the depletion of ATP can direct cells between apoptotic and necrotic pathways [99]. During normal apoptosis, PARP1 is cleaved by caspases; the role of these cleaved fragments play is not fully understood [[A57]]. PARP1 cleavage helps prevent energy depletion (NAD + and ATP) in response to severe DNA damage; the extreme loss of NAD + triggers necrosis by reducing cellular ability to synthesize ATP [[A58]] [100]. Cells with severe DNA damage die from necrosis because they are not able to switch away from the necrotic pathway since the kinetics of NAD + depletion are faster than those of PARP1 cleavage [2]. Rapid depletion of NAD + levels by PARP1 reduces SIRT1 activity and inhibits the capability of SIRT1 to deacetylate its targets respond to genotoxic stress [101]. PARP1 has also been implicated in caspase-independent apoptosis, where its activation leads to apoptosis-inducing factor (AIF) release from the mitochondria, which induces nuclear chromatin fragmentation [[A59]] [102].

#### Circadian rhythms

**Figure 6:**
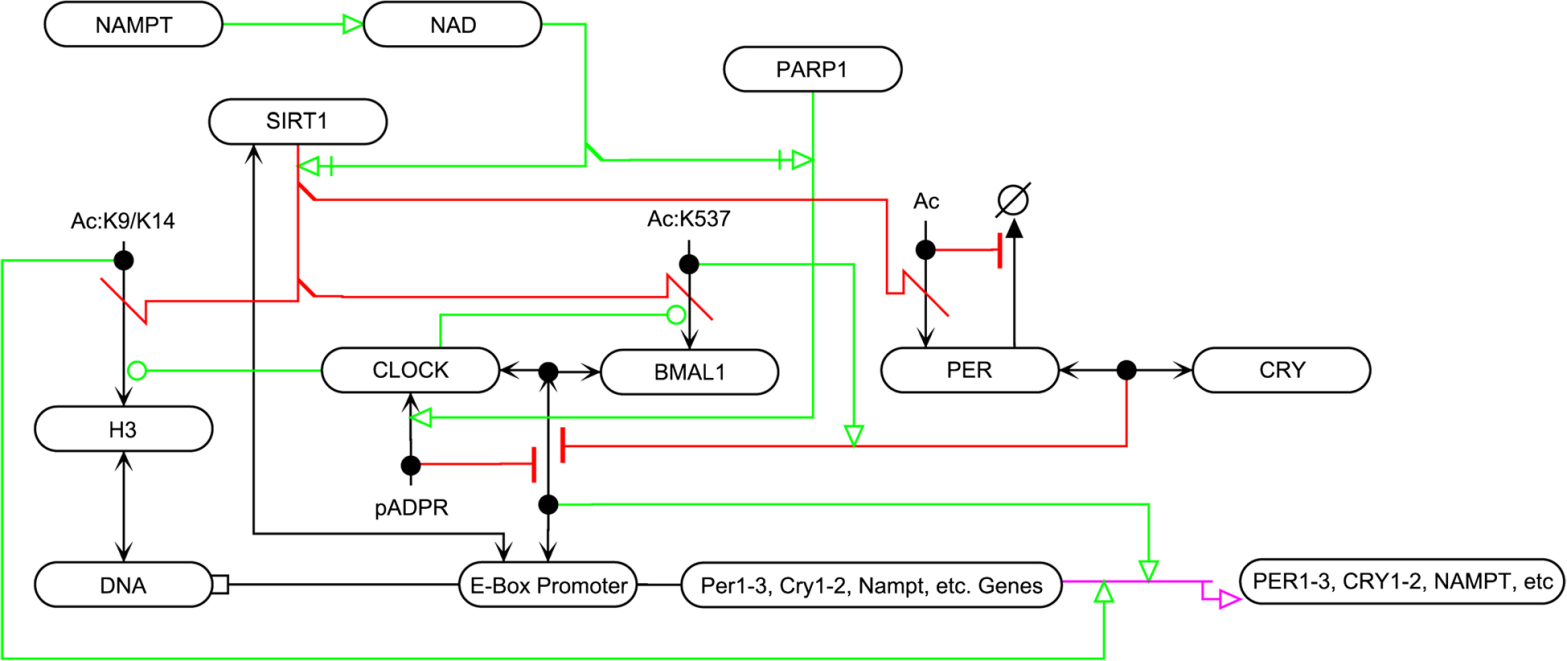
Interactions of SIRT1 and PARP1 with circadian clock components.

Recent SIRT1 and PARP1 research has uncovered roles for the two proteins in circadian rhythms creating the possibility for novel interconnections between metabolism, DNA repair, and circadian rhythms (Figure 6). The core circadian machinery involves a transactivating CLOCK/BMAL1 heterodimer, which induces the transcription of a large number of genes, including the cryptochrome (CRY1 and CRY2) and period (PER1, PER2, PER3) genes that form a complex that leads to a negative feedback loop suppressing CLOCK/BMAL1-mediated transcription [[A60-A62]]. Several studies have shown that disruptions in core circadian interactions can lead to alterations in DDR; reviewed in [103].

SIRT1 deacetylates BMAL1 at K537 destabilizing the interaction between CRY and BMAL1 [[A63]] [104,105]. CLOCK possesses acetyltransferase activity that regulates the transcriptional activity of CLOCK/BMAL1 [106] and is capable of acetylating some of the same locations that SIRT1 deacetylates: H3K9, H3K14, and BMAL1 at K537 [[A64]] [57,105]. SIRT1 has also been shown to deacetylate PER2 destabilizing the protein [[A65]]; it has been hypothesized that acetylation of PER2 at lysine residues prevents their ubiquitination [107]. This presents a dual control mechanism for SIRT1 in the circadian clock where it is capable of balancing transcription through chromatin condensation, but also by disrupting the ability for CRY and PER2 to repress CLOCK/BMAL1 activity. SIRT1 is involved in NAMPT transcriptional regulation, which is under circadian control causing NAD + levels to oscillate as a consequence of NAMPT level oscillation [48,108]. Additionally, it has been shown that PARP1 has rhythmic activity influenced by feeding patterns though further work is necessary to understand the underlying molecular mechanism. PARP1 is capable of ADP-ribosylating CLOCK in a circadian manner disrupting the association between the BMAL1/CLOCK heterodimer and its targets [[A66]] [109]. It remains to be determined whether a regulatory effect exists between SIRT1 or PARP1 and the circadian components during DNA damage.

#### Interactions with other family members

While the focus of this review has been on the inter-relationships that exist between SIRT1 and PARP1, there is growing evidence that SIRT1 has the capability of interacting with other members of the PARP family of proteins and similarly that PARP1 is capable of interacting with multiple sirtuins. Here we present three cases of these interactions; these interactions range include both direct modifications, as well as transcriptional regulation. First, SIRT6, one of the nuclear sirtuins, plays a role in promoting DNA damage repair by binding and activating PARP1 by mono-ADP-ribosylating PARP1 triggering its auto-ADP-ribosylation activity [[A67]] [110]. Next, the second PARP family member, PARP2, has been shown to inhibit the transcription of SIRT1; the deletion of PARP2 increases overall levels of SIRT1 activity without having to target NAD + levels directly [[A68]] [46]. This finding indicates that inhibitors of PARP proteins may be capable of increasing SIRT1 activity not only via the inhibition of NAD + consumption by PARP family members, but also through the removal of transcriptional inhibition. Lastly, shown recently is that PARP1 increases levels of mitochondrial SIRT3 and that SIRT3 can continue to function under stress conditions because mitochondrial NAD + levels are maintained in the conditions produced by either treatment with methylnitronitrosoguanidine (MNNG), a carcinogen, or N-methyl-D-aspartate (NMDA), a neuronal stressor, even as cytosolic levels of NAD + are depleted by PARP1 [111,112]. This study by Kim et al., did not observe a similar change in SIRT1 protein levels or the expression levels of the other mitochondrial sirtuins, SIRT4 and SIRT5.

## Conclusions

Cells respond to DNA damage through coordinated pathways that arrest the cell cycle and repair the damage, and in the presence of severe damage trigger cell death. Both SIRT1 and PARP1 play an intimate role in the regulation of genomic stability, and continued work is necessary for the understanding of the specific contexts in which modulators of SIRT1 and PARP1 activity may be appropriate as therapeutics for cancer and metabolic disorders. The regulation of SIRT1 and PARP1 is controlled by a variety of stimuli, including metabolic, circadian, and genotoxic. Understanding how each of these stimuli affects the regulatory network of these two proteins is vital.

The work of the past several years has trended towards an understanding of the diverse regulatory network surrounding these two proteins, the effects of local levels of their common substrate, NAD+, and the co-regulation of and by the two proteins. There continue to be several unexplored areas. For example, understanding the nature of competitive regulation of acetylation and PAR at a single substrate residue on a single molecule, since both can target lysine residues, remains an open area of research. Another interesting avenue of research remaining to be thoroughly explored is the alteration of the reviewed pathways as an organism ages. Studies have shown age-dependent increases in DNA damage can lead to NAD + depletion [113,114]. In human tissue samples, Massudi et al. found an age-related positive correlation in PARP1 activity in males and negative correlations with SIRT1 activity in males and NAD + levels in both males and females that starts to shed light on the role of these two proteins in the presence of accumulating DNA damage with age [114]. In a related study, Chang et al. showed a reduction in SIRT1 levels over time resulting in alterations in circadian oscillations in mice as they age [115]. Studies such as these suggest that the regulatory network surrounding SIRT1 and PARP1 may undergo large-scale changes over an organism’s lifespan; currently, we do not know the extent of these changes. Additionally, there is a deficit in our knowledge with respect to the influence that DNA damage response may have on circadian regulation and vice versa and the roles that SIRT1 and PARP1 may play. And while this review has focused on evidence of the interactions between the better studied members of their respective protein families, SIRT1 and PARP1, additional work is necessary in our understanding of the roles of the other members of the two protein families and their unique properties and interactions that may play integral roles in the progression of DDR, as well as other processes.

## Competing interests

The authors declare that they have no competing interests.

## Authors' contributions

AL wrote the manuscript and drew and annotated the supplemental MIM. MIA and KWK edited the manuscript and MIM. All authors have read and approved the final manuscript.

## Supplementary Material

Additional file 1 **SIRT1/PARP1 MIM GPML (GenMAPP Pathway Markup Language) File.** The SIRT1/PARP1 MIM as a machine-readable file that can be read and searched using PathVisio-MIM ( http://discover.nci.nih.gov/mim/mim_pathvisio.html). Click here for file

Additional file 2 **SIRT1/PARP1 MIM Annotations Excel File.** Table containing annotations for all interactions shown in the SIRT1/PARP1 MIM. Click here for file
